# Adult‐Onset IgA Vasculitis in Early Pregnancy Following Rhinovirus/Enterovirus and Streptococcal Infection: A Case Report and Review of Literature

**DOI:** 10.1155/carm/1612186

**Published:** 2026-08-27

**Authors:** Isaac Kah Siang Ng, Charisse Wan Ning Loh, Wilson Guo Wei Goh, Elaine Ah Gi Lo, Kee Fong Phang, Jiacai Cho, Kong Bing Tan, May Shuen Tang

**Affiliations:** ^1^ Division of Rheumatology and Allergy, Department of Medicine, National University Hospital, Kent Ridge, Singapore, nuh.com.sg; ^2^ Division of Infectious Diseases, Department of Medicine, National University Hospital, Kent Ridge, Singapore, nuh.com.sg; ^3^ Department of Pharmacy, National University Hospital, Kent Ridge, Singapore, nuh.com.sg; ^4^ Division of Rheumatology, Department of Medicine, Alexandra Hospital, Queenstown, Singapore, ah.com.sg; ^5^ Department of Pathology, National University Hospital, Kent Ridge, Singapore, nuh.com.sg

## Abstract

IgA vasculitis (IgAV) is an immune complex‐mediated systemic small‐vessel vasculitis that is mostly seen in children and triggered by respiratory or gastrointestinal tract infections. Rarely, it may occur in adults, with rare cases developing during pregnancy. We herein described a unique case of a young lady who developed skin rashes and inflammatory joint pains secondary to IgAV preceded by two bouts of respiratory tract infection (*Streptococcal* throat infection and rhinovirus/enterovirus infection). She was also found to be in early pregnancy (2 weeks gestational age) at onset of IgAV. She was subsequently managed carefully by the rheumatologist and obstetrician with a tapering course of corticosteroid monotherapy without initiation of steroid‐sparing immunosuppressant. This case highlights the unique entity of managing adult IgAV in pregnancy, for which therapeutic considerations require balancing the degree of immunosuppression and the need to avoid active rheumatic disease which both pose significant risks in pregnancy.

## 1. Introduction

IgA vasculitis (IgAV) is an immune complex‐mediated small‐vessel vasculitis, characterised by purpuric vasculitic rashes (typically distributed over the lower limbs and buttocks), arthritis, gastrointestinal manifestations and glomerulonephritis [1, 2]. It is most commonly diagnosed in childhood, with 90% of cases occurring between 2 and 10 years of age [2]. The pathophysiology of IgAV is postulated to involve environmental triggers (infections, medications and vaccinations) that provoke abnormal IgA‐mediated immune reactions in genetically predisposed hosts [1–3]. Less commonly IgAV can present in adults, who tend to have a higher prevalence and more severe renal manifestations than their paediatric counterparts [1]. Besides the aforementioned triggers, pregnancy has rarely been described as a precipitant of IgAV in adult females of reproductive age [4].

We herein present a case of a 34‐year‐old female patient who was diagnosed with IgAV that was temporally preceded by enterovirus/rhinovirus and *Streptococcal* throat infections, and simultaneously occurring in early pregnancy. She was successfully managed with tapering steroid monotherapy with an uneventful pregnancy and no IgAV flare‐ups. Given the rarity of new‐onset IgAV during pregnancy, we further discussed the immunosuppressive management considerations in this patient population and reviewed other described cases in existing literature.

## 2. Clinical Vignette

**FIGURE 1:**
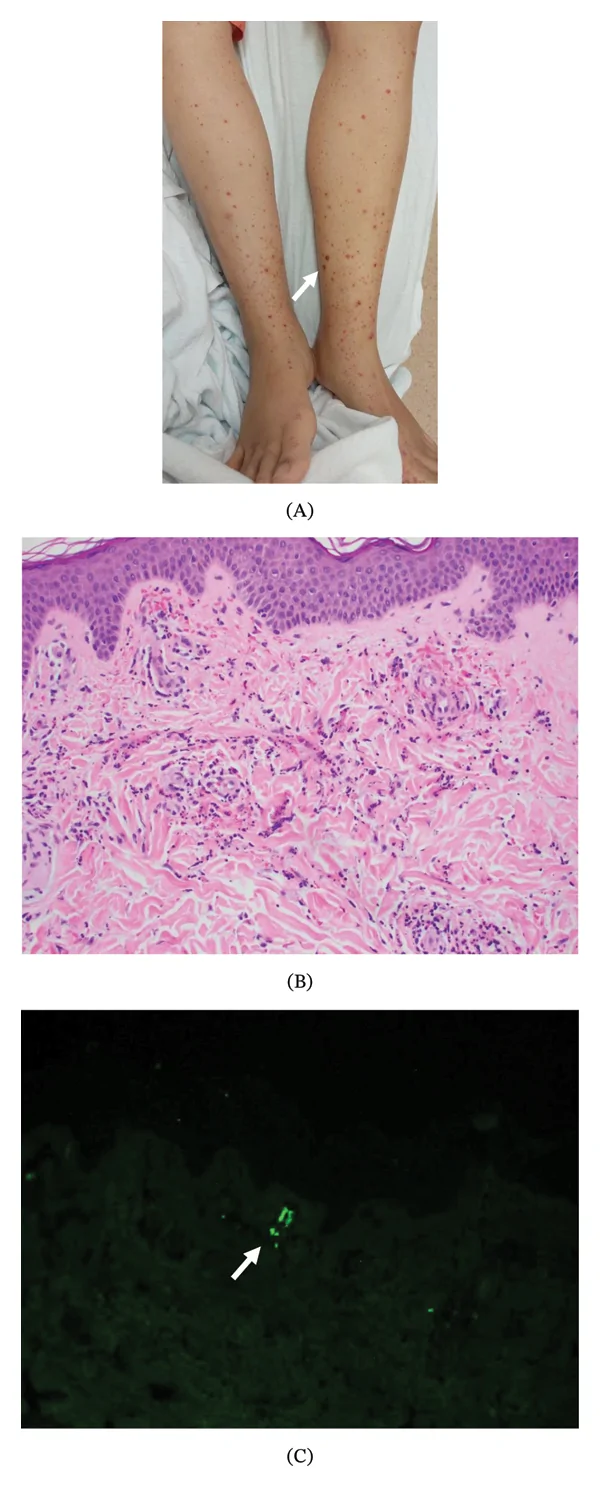
(A) Clinical photo showing scattered purpuric papules (arrow) distributed over bilateral anterior shins and dorsum of the feet. (B) Histopathology slide of skin biopsy showing dermal perivascular and interstitial infiltrate of inflammatory cells with nuclear dust material and red blood cell extravasation (Haematoxylin and eosin stain, original magnification × 200). (C) Immunofluorescence of skin biopsy sample showing focal deposits of IgA (1+) (white arrow) around small dermal blood vessels (original magnification × 200).

A 34‐year‐old healthy Chinese female presented to the hospital with a 5‐day history of nonpruritic, nontender bilateral lower limb purpuric rashes, after two recent bouts of upper respiratory tract infection in the preceding month. She reported no fever, weight loss, joint pain, abdominal or neurological symptoms. On arrival, she was afebrile with normal blood pressure (108/65 mmHg). Examination revealed palpable purpuric papules over bilateral shins and dorsum of feet (Figure 1A) and pharyngeal injection, but with normal systemic examination. Three days postadmission, she also developed severe inflammatory polyarthritis of the metacarpophalangeal/proximal interphalangeal joints, left knee and bilateral ankles.

Initial laboratory investigations (Table 1) revealed normal full blood count, renal and liver function tests, and elevated C‐reactive protein (CRP; 31.8 mg/L) and erythrocyte sedimentation rate (ESR; 22 mm/h). Her urinalysis showed very mild microscopic haematuria, no significant proteinuria, and the initial urine pregnancy test was negative. While COVID‐19 antigen rapid testing was negative, a nasal swab for respiratory pathogens detected human rhinovirus/enterovirus. Antistreptolysin O titres were also elevated at 800 IU/mL. Hepatitis B/C and human immunodeficiency virus screen were negative. Autoimmune studies comprising antinuclear antibody, antidouble‐stranded DNA, complement C3/C4, antiextractable nuclear antigens and antineutrophilic cytoplasmic antibodies were negative. Skin biopsy of left foot purpuric rash revealed neutrophilic‐predominant perivascular inflammatory infiltrates, with nuclear dusts, extravasated erythrocytes (Figure 1B) and direct immunofluorescence findings of focal deposits of IgA (1+) (Figure 1C).

**TABLE 1:** Pertinent laboratory investigations.

Investigations	Results	Reference range	Units
*Full blood count (at diagnosis)*			

White blood cell	9.47	4.3–10.4	x 10^9^/L

Haemoglobin	12.8	13.1–16.8	g/L

Platelets	268	150–410	x 10^9^/L

*Inflammatory markers*			

C‐reactive protein	**31.8**	< 5	mg/L

Erythrocyte sedimentation rate	**22**	< 15	mm/h

*Renal panel*			

Sodium	140	135–145	mmol/L

Potassium	4.1	3.5–5.2	mmol/L

Chloride	109	95–110	mmol/L

Bicarbonate	25	22–32	mmol/L

Urea	3.4	3.2–7.4	mmol/L

Creatinine	46	60–110	μmol/L

*Liver function tests*			

Albumin	51	35–52	g/L

Bilirubin (total)	7	1–20	μmol/L

Alanine transaminase	19	6–40	U/L

Aspartate transaminase	23	6–35	U/L

Alkaline phosphatase	79	50–116	U/L

*Infective workup*			

COVID‐19 Antigen Rapid test (nasal swab)	Negative	—	—

Respiratory pathogens panel (nasal swab)	Human rhinovirus/enterovirus detected	—	—

Dengue Duo (NS1, IgM, IgG)	Negative	—	—

Hepatitis B screen (HBsAg, anti‐HBc, anti‐HBs)	HBsAg negative, anti‐HBc negative, anti‐HBs antibody positive (Immune status)	—	—

Hepatitis C screen (anti‐HCV antibody)	Negative	—	—

HIV screen	Negative		

Antistreptolysin O titre	800	< 200	IU/mL

*Autoimmune studies*			

Antinuclear antibody	< 1:80	< 1:80	titre

Antidouble‐stranded DNA	< 10	< 100	IU/mL

Complement 3	112	82–185	mg/dL

Complement 4	28	15–53	mg/dL

Antiextractable nuclear antigens	Negative	Negative	—

Antineutrophil cytoplasmic antibodies (anti‐MPO, anti‐PR3)	Anti‐MPO < 2	Anti‐MPO < 20	U/mL
	Anti‐PR3 < 5	Anti‐PR3 < 20	

*Urine tests (at diagnosis)*			

Urine formed element microscopic examination	Red blood cell—**3**	Red blood cell ≤ 2	Cells/HPF
	White blood cell—**5**	White blood cell ≤ 2	
	Epithelial cells—present	Epithelial cells – not seen	

Urine red cell morphology	Dysmorphic 10%, isomorphic 90%	—	—

Urine protein : creatinine ratio	8	< 20	mg/mmol

Urine pregnancy test	Negative	—	—

*Repeat urine pregnancy test (2 weeks after rashes onset)*			

Urine pregnancy test	Positive	—	—

*Interval renal function and urinalyses (3 weeks posttreatment)*			

Serum creatinine	61	60–110	μmol/L

Urine formed element microscopic examination	Red blood cell < 1	Red blood cell ≤ 2	Cells/HPF
	White blood cell < 1	White blood cell ≤ 2	
	Epithelial cells—present	Epithelial cells—not seen	

Urine dipstick	Nil protein	—	—

*Interval renal function and urinalyses (6 weeks posttreatment)*			

Serum creatinine	49	60–110	μmol/L

Urine formed element microscopic examination	Red blood cell—2	Red blood cell ≤ 2	Cells/HPF
	White blood cell—1	White blood cell ≤ 2	
	Epithelial cells—present	Epithelial cells—not seen	

Urine protein : creatinine ratio	Undetectable	< 20	mg/mmol

*Interval renal function and urinalyses (12 weeks posttreatment)*			

Serum creatinine	45	60–110	μmol/L

Urine formed element microscopic examination	Red blood cell—**3**	Red blood cell ≤ 2	Cells/HPF
	White blood cell—1	White blood cell ≤ 2	
	Epithelial cells—present	Epithelial cells—not seen	

Urine protein : creatinine ratio	7	< 20	mg/mmol

*Interval renal function and urinalyses (22 weeks posttreatment)*			

Serum creatinine	44	60–110	μmol/L

Urine formed element microscopic examination	Red blood cell—**3**	Red blood cell ≤ 2	Cells/HPF
	White blood cell—4	White blood cell ≤ 2	
	Epithelial cells—present	Epithelial cells – not seen	

Urine protein : creatinine ratio	9	< 20	mg/mmol

*Note:* Bold indicates abnormal or pertinent results.

The patient was diagnosed with IgAV (with cutaneous and joint manifestations) likely precipitated by rhinovirus/enterovirus and streptococcal coinfections, and occurring in early pregnancy. She was started on prednisolone initially at 10 mg/day, but she continued to have worsening rashes and joint symptoms, necessitating uptitration to an initial maximum dose of 30 mg/day (0.5 mg/kg/day). She was also given a 10‐day course of amoxicillin for possible *Streptococcal* throat infection.

Two weeks posttreatment initiation, the patient repeated a urine pregnancy test due to missed menstrual cycle, which turned positive. Foetal ultrasound at the obstetrics clinic confirmed a viable pregnancy. Her calculated gestational age at the time of onset of vasculitic rashes would have been 2 weeks.

Given the possibility of IgAV flare‐up during pregnancy, steroid‐sparing immunosuppressive options were discussed with the patient including colchicine, azathioprine, cyclosporine and tacrolimus which were safe for use in pregnancy; however, she declined and opted for a gradual steroid‐tapering regimen with close clinical monitoring.

At 3 weeks follow‐up after treatment initiation, her joint symptoms have completely resolved, rashes have largely subsided, and her CRP and ESR normalised to < 0.4 mg/L and 6 mm/h, respectively. Her renal function and urinalyses were normal on interval assessments at 3, 6, 12 and 22 weeks posttreatment initiation. Her steroid was subsequently tapered by 5 mg every week initially, until 10 mg/day, following which it was tapered by 1 mg every 2 weeks until 2 mg/day, at which point decision was made to stop the steroids completely given persistent disease remission (Table 2). Her steroid treatment course lasted for 22 weeks, and at the point of discontinuation of steroids, she was at 23 weeks of gestation. At the time of writing, she remains on active follow‐up with rheumatology and her pregnancy has been progressing well at 32 weeks gestational age with no signs of IgAV flare.

**TABLE 2:** Treatment course and steroid‐tapering regimen.

Time since diagnosis (weeks) (patient was formally diagnosed 1 week after onset of rashes)	Gestational age (weeks)	Prednisolone dose (mg/day)
0	3	10
1	4	30
2	5	25
3	6	20
4	7	15
5	8	10
7	10	9
9	12	8
11	14	7
13	16	6
15	18	5
17	20	4
19	22	3
21	24	2
22	25	0

## 3. Discussion

IgAV is an immune complex‐mediated small vessel vasculitis that is most commonly seen in childhood, with 90% occurring between 3 and 15 years of age [3]. However, it can infrequently occur in adults, in whom there is a higher occurrence of nephritis (45%–85%), with poorer renal outcomes [5, 6]. Similar to paediatric cases, majority of adult‐onset cases are triggered by a preceding infection, which could be a viral upper respiratory tract infection or bacterial (e.g., skin or gastrointestinal tract) infection [7]. Less commonly, drugs, vaccines, malignancy, and pregnancy have also been implicated as precipitants [3, 8] while most cases of IgAV run a monophasic course—approximately one‐third of cases recur/relapse [1, 7]. A recent multicentre cohort study identified fever, abdominal purpura and joint involvement as predictors of relapse/recurrence in adult IgAV, while steroid use and postbacterial aetiology appeared to be protective [7].

Treatment of mild cases of skin or joint‐limited IgAV is largely supportive, with the use of topical corticosteroids and nonsteroidal anti‐inflammatory drugs. However, systemic steroids are considered in severe skin/joint disease, with high doses (1 mg/kg/day of prednisolone or equivalent) necessary for patients with major end‐organ involvement (e.g., renal, gut, cerebral and pulmonary vasculitis). In adult‐onset IgAV occurring in pregnancy, however, steroid exposure should be minimised due to the risk of hypertension/pre‐eclampsia, gestational diabetes, infections, preterm births and intrauterine growth restriction [9]. Yet, this must be balanced with pregnancy risks incurred if the underlying systemic vasculitis is poorly controlled or flares up. Nonsteroidal immunosuppressive treatments in IgAV which are safe for pregnancy include hydroxychloroquine, azathioprine, cyclosporine or colchicine if benefits outweigh potential risks, while mycophenolate mofetil and cyclophosphamide must be avoided [10]. Importantly, close monitoring especially regular assessments of renal function and urinalysis is necessary for early detection of glomerulonephritis. In general, for patients with normal findings at diagnosis, assessments may be repeated every 2 weeks in the first month, followed by every 2 months in the subsequent 6–12 months, and annually thereafter if labs remain normal [11].

Literature review of studies on pregnant patients with pre‐existing IgAV generally shows favourable maternal and foetal outcomes. Sangle et al. studied the pregnancy outcomes of 29 pregnant females with systemic vasculitis, of which one had pre‐existing IgAV [12]. This patient developed a cutaneous flare during pregnancy, but her pregnancy was otherwise uncomplicated [12]. Nossent et al. performed a cohort study of pregnancy outcomes in 247 pregnant patients with prior history of IgAV and 556 pregnancies in control in Western Australia and found that none of the IgAV patients had disease flare during pregnancy, although there were 43 unsuccessful pregnancies (17.4%) and 17 preterm deliveries out of the 204 live births (8.3%) [13]. The authors also reported that females with IgAV had higher odds of spontaneous abortion, preterm delivery and gestational hypertension than the control group [13]. Beca et al.’s cohort study on the maternal/foetal outcomes of pregnancies in females with systemic vasculitis included three patients with pre‐existing IgAV [14]. While all the patients had successful deliveries, 1 developed IgAV renal flare in the second trimester, developed pre‐eclampsia, and had foetal complications of intrauterine growth restriction/small for gestational age and low birth weight, 1 had antepartum haemorrhage due to placenta previa and preterm birth with no IgAV flare during pregnancy, and the last patient had completely event‐free pregnancy [14].

However, data on pregnancy and clinical outcomes of patients who developed new‐onset IgAV during pregnancy are sparse owing to its rarity. Besse et al. performed a retrospective case–control study in France comparing pregnancy outcomes in 26 patients with pre‐existing IgAV, 15 patients who developed IgAV post‐pregnancy and 52 pregnancies in healthy women [15]. They found that gestational hypertension and pre‐eclampsia were more frequent in those with pre‐existing IgAV compared to those with postpregnancy IgAV and healthy controls, and this was particularly prevalent in those with pre‐existing renal impairment [15]. None of the IgAV patients had disease flare during pregnancy [15]. Of those which reported immunosuppressive treatment use for treatment of IgAV during pregnancy, the therapeutics given were corticosteroids, colchicine, dapsone and azathioprine [12, 14, 15]. Separately, there have only been several individual case reports which describe new‐onset IgAV in pregnancy. There were three cases first described in the 1980s, which were supportively managed, with one successful pregnancy [16], one pregnancy termination [17] and one spontaneous abortion [4]. However, there was generally lack of available details on these cases. More recent cases from 2002 to 2018 described successful, uncomplicated pregnancies [18–22], with either supportive management for milder cases [18–20], or tapering corticosteroid monotherapy [18, 21, 22]. Mild cutaneous flares were described in two patients during pregnancy [18], but none had severe vasculitic manifestations [18–22].

In conclusion, adult IgAV that occurs during pregnancy is a rare clinical entity, for which management considerations require the selection of appropriate immunosuppressive treatments that can adequately achieve disease control while minimising treatment‐associated risks to maternal and foetal health. Further multicentre studies and pregnancy registries would be helpful to better characterise the clinical manifestations, triggers, prognostic factors and optimal therapeutic approaches in this unique patient population.

## Author Contributions

Isaac Kah Siang Ng conceived the study idea and wrote the manuscript. Charisse Wan Ning Loh, Wilson Guo Wei Goh, Elaine Ah Gi Lo, Kee Fong Phang, Jiacai Cho, Kong Bing Tan and May Shuen Tang critically reviewed and edited the manuscript. Isaac Kah Siang Ng, Charisse Wan Ning Loh, Kee Fong Phang, Jiacai Cho and May Shuen Tang were directly involved in patient care.

## Funding

The authors have nothing to report.

## Consent

Written informed patient consent has been obtained.

## Conflicts of Interest

The authors declare no conflicts of interest.

## Data Availability

Data of the case (anonymised) are available for journal review upon reasonable request made to the corresponding author.

## References

[1] Gage A. , Pepper R. J. , Marro J. , Salama A. D. , and Oni L. , IgA Vasculitis Across the Ages: Is it Time for a Precision Medicine Approach?, ACR Open Rheumatology. (2025) 7, no. 9, 10.1002/acr2.70083. PMC12426765 40937635

[2] Leung A. K. C. , Barankin B. , and Leong K. F. , Henoch-Schönlein Purpura in Children: An Updated Review, Current Pediatric Reviews. (2020) 16, no. 4, 265–276, 10.2174/1573396316666200508104708. 32384035

[3] Parums D. V. , A Review of IgA Vasculitis (Henoch-Schönlein Purpura) Past, Present, and Future, Medical Science Monitor. (2024) 30, 10.12659/msm.943912. PMC10832303 38281080

[4] Siroty R. , Henoch-Schonlein Purpura in Pregnancy, Journal of the Medical Society of New Jersey. (1985) 82, 535–536. 3861874

[5] Sugino H. , Sawada Y. , and Nakamura M. , IgA Vasculitis: Etiology, Treatment, Biomarkers and Epigenetic Changes, International Journal of Molecular Sciences. (2021) 22, no. 14, 10.3390/ijms22147538. PMC8307949 34299162

[6] Tonelli M. , Targeting the Pathogenesis of IgA Nephropathy—A New Treatment Approach?, New England Journal of Medicine. (2026) 394, no. 7, 712–713, 10.1056/nejme2518418. 41671487

[7] Lutz W. , Hua C. , Mariotti E. B. et al., Risk Factors for Recurrence or Relapse After a First Episode of Adult IgA Vasculitis: A Multicenter Retrospective Study, Journal of the American Academy of Dermatology. (2025) 9622, no. 25, 02956–1. 10.1016/j.jaad.2025.10.009 41061973

[8] Tayabali S. , Andersen K. , and Yoong W. , Diagnosis and Management of Henoch-Schönlein Purpura in Pregnancy: A Review of the Literature, Archives of Gynecology and Obstetrics. (2012) 286, no. 4, 825–829, 10.1007/s00404-012-2468-2. 22865032

[9] Pofi R. and Tomlinson J. W. , Glucocorticoids in Pregnancy, Obstetric Medicine. (2020) 13, no. 2, 62–69, 10.1177/1753495x19847832. 32714437 PMC7359660

[10] Russell M. D. , Dey M. , Flint J. et al., British Society for Rheumatology Guideline on Prescribing Drugs in Pregnancy and Breastfeeding: Immunomodulatory Anti-Rheumatic Drugs and Corticosteroids, Rheumatology. (2023) 62, no. 4, E48–E88, 10.1093/rheumatology/keac551. 36318966 PMC10070073

[11] Stichert V. R. , Santoso B. , Winters B. G. et al., Updates on the Diagnosis, Prognosis, and Management of IgA Vasculitis in Adults: A Narrative Review, Journal of the American Academy of Dermatology. (2026) 9622, no. 26, 00170–00172. 10.1016/j.jaad.2026.02.007 41655840

[12] Sangle S. R. , Vounotrypidis P. , Briley A. et al., Pregnancy Outcome in Patients With Systemic Vasculitis: A Single-Centre Matched Case-Control Study, Rheumatology. (2015) 54, no. 9, 1582–1586, 10.1093/rheumatology/kev018. 25832613

[13] Nossent J. , Raymond W. , Keen H. , Inderjeeth C. , and Preen D. , Pregnancy Outcomes in Women With a History of Immunoglobulin A Vasculitis, Rheumatology. (2019) 58, no. 5, 884–888, 10.1093/rheumatology/key408. 30590848

[14] Beça S. , Alba M. A. , Hernández-Rodríguez J. et al., Maternal and Fetal Outcomes of Pregnancy in Women With Primary Systemic Vasculitis: A Single-Center Cohort Study of 20 Patients and 30 Pregnancies, Seminars in Arthritis and Rheumatism. (2024) 66, 10.1016/j.semarthrit.2024.152412. 38387195

[15] Besse M. C. , Perrotin F. , Aouba A. et al., Pregnancy Outcome in Patients With a Medical History of Immunoglobulin A Vasculitis: A Case–Control Study, Scandinavian Journal of Rheumatology. (2024) 53, no. 1, 36–43, 10.1080/03009742.2023.2226518. 37439394

[16] McCoy M. , Henoch-Schönlein Purpura and Pregnancy, American Journal of Obstetrics and Gynecology. (1981) 141, no. 4, 469–470, 10.1016/0002-9378(81)90616-5. 7282834

[17] Wilks R. , Abdella T. , and Alfano C. , Henoch-Schonlein Purpura Associated With Eclampsia. A Case Report, Journal of Reproductive Medicine. (1993) 38, no. 8, 645–646. 8410873

[18] Cummins D. , Mimouni D. , Rencic A. , Kouba D. , and Nousari C. , Henoch-Schönlein Purpura in Pregnancy, British Journal of Dermatology. (2003) 149, no. 6, 1282–1285, 10.1111/j.1365-2133.2003.05671.x. 14674910

[19] Côté J. M. , Meunier R. S. , Tremblay J. A. , Weber F. , and Mahone M. , Henoch-Schonlein Purpura in Pregnancy: A Case Report, Obstetric Medicine. (2018) 11, no. 4, 195–197, 10.1177/1753495x17745391. 30574183 PMC6295767

[20] Djakovic I. , Butorac D. , Vucicevic Z. , Kosec V. , Kuna A. T. , and Lugović-Mihić L. , Henoch-Schönlein Purpura in the Third Trimester of Pregnancy, Biochemical Medicine. (2018) 28, no. 1, 10.11613/bm.2018.010801. PMC5806616 29472804

[21] Koizumi M. , Hagino D. , Fukuyama C. et al., Schönlein-Henoch Purpura During Pregnancy: Case Report and Review of the Literature, Journal of Obstetrics and Gynaecology Research. (2004) 30, no. 1, 37–41, 10.1111/j.1341-8076.2004.00153.x. 14718019

[22] Feldmann R. , Rieger W. , Sator P. G. , Gschnait F. , and Breier F. , Schönlein-Henoch Purpura During Pregnancy With Successful Outcome for Mother and Newborn, BioMed Central Dermatology. (2002) 2, 10.1186/1471-5945-2-1. PMC65680 11866865

